# Role of Monoaminergic System in the Etiology of Olive Oil Induced Antidepressant and Anxiolytic Effects in Rats

**DOI:** 10.1155/2013/615685

**Published:** 2013-07-10

**Authors:** Tahira Perveen, Bilal Moiz Hashmi, Saida Haider, Saiqa Tabassum, Sadia Saleem, Munnawar Ahmed Siddiqui

**Affiliations:** Neurochemistry and Biochemical Neuropharmacology Research Unit, Department of Biochemistry, University of Karachi, Karachi 75270, Pakistan

## Abstract

Olive oil is the major component of the Mediterranean diet and has rich history of nutritional and medicinal uses. In the present study, the antidepressant and anxiolytic effects and their neurochemical basis following repeated administration of extravirgin olive oil were monitored. Male albino Wistar rats were used during study. Animals of test group were given olive oil orally at the dose of 0.25 mL/kg daily for 4 weeks. Control rats received equal volume of water. Elevated-plus maze (EPM) test and forced swim test (FST) were performed for the assessment of anxiety and depression like symptoms. An increase in time spent in open arm in EPM and increased struggling time in FST following long-term administration of olive oil indicate that olive oil has anxiolytic and antidepressant properties. Neurochemical results showed that repeated administration of olive oil decreased the levels of brain 5-HT (5-hydroxytryptamine), 5-HIAA (5-hydroxyindoleacetic acid), and levels of DA (dopamine); however, levels of DA metabolite HVA (homovalinic acid) were increased. Hence, present findings suggest that olive oil has neuroprotective effects. It reduces behavioral deficits via altering 5-HT and DA metabolism. So it could be used as a therapeutic substance for the treatment of depression and anxiety.

## 1. Introduction

Olive (*Olea europaea*) is in the family Oleaceae that is used throughout the world especially in the Mediterranean region. It is full of nutrients and vitamins. Extravirgin olive oil is derived from the first pressing of the olives. It has the most delicate flavor and antioxidant benefits [1]. Olive oil is rich in monounsaturated fatty acids (MUFAs) and has excellent health benefits [2]. Olive oil is composed mainly of mixed triglyceride esters of oleic acid, palmitic acid, and other fatty acids, along with the traces of squalene (up to 0.7%) and sterols (about 0.2%; phytosterol & tocosterols). It also contains group of antioxidants that are esters of tyrosol and hydroxytyrosols, including oleocanthal, oleuropein, vitamin E, and carotenoids. Oleuropein is a chemical that prevents the oxidation of LDL (low density lipoproteins) particles [3].

Evidence from epidemiological studies suggests that a higher proportion of monounsaturated fats in the diet are linked with a reduction in the risk of coronary heart disease [4]. It has been reported that olive oil consumption has favorable effects on cholesterol regulation and LDL cholesterol oxidation and it also exerts antiplatelet [5], anti-inflammatory, antithrombotic, and antihypertensive as well as vasodilatory effects on both animals and humans [6]. Phenolic compounds, oleic acid, and tocopherols are also thought to be related to some of these beneficial effects [7]. Olive oil could possibly be a chemopreventive agent for peptic ulcer or gastric cancer [8] and was found to reduce oxidative damage to DNA and RNA, which may be a factor in preventing cancer [9]. Olive oil is rich in omega 3 and omega 6 fatty acids which help to improve memory [10] and support the CNS by aiding nerves to do proper function and increases serotonin levels [11]. Evidence has shown that high MUFA intake appeared to be protective against age-related cognitive decline and helps to aid the memory [10]. Olive oil also helps to build a more healthy balance between omega-6 fats and omega-3 fats [12]. It has been reported that omega-3 fatty acid, docosahexaenoic acid (DHA), plays an adaptive role during stress and shows a significant reduction in perceived stress [13] by increasing secretion of adrenal corticosterone in rats loaded with single repetitive stress [14].

Oleuropein derivatives found in olive oil especially hydroxytyrosol have been shown to have protective effects not only against markers associated with antherogenic process [7, 15, 16] but has an antioxidant capacity higher than that of other known antioxidants such as vitamins E and C [17]. In addition to this, an anxiety lowering effect with reduced step-through latency in light dark box test was also found in old animals that the effect was due to the decreased glutathione reductase activity and expression in the brain [18]. Studies have also reported that treatment with olive extract resulted in a significant decline in the immobility time as well as hyperalgesia [19].

It is evident that oleamide induced increase in slow-wave sleep, a decrease in wakefulness and sleep latency, was prevented by 5-HT reuptake inhibitors such as fluoxetine and by agonists at 5-HT1A receptors such as buspirone or 8-OH-DPAT [20]. Oleamide causes a potentiation of 5-HT elicited inositol phosphate formation mediated by the 5-HT2A receptors but inhibited the effects of 5-HT on cAMP production mediated by 5-HT7 receptor [21]. Hence, extravirgin olive oil is becoming more important in daily diets nowadays, particularly in Mediterranean diet, due to its beneficial health effects on human health. Therefore, the present study was designed to investigate the neuroprotective effects of extra-virgin olive oil in rats and to elucidate the neurochemical changes associated with it.

## 2. Materials and Methods

### 2.1. Animals

Male albino Wistar rats weighing from 140–280 g were purchased from Animal House of HEJ Research Institute of Chemistry, University of Karachi, Pakistan. The animals were caged separately in plastic cages at room temperature of 22 ± 2°C with free access to tap water and cubes of standard rodent diet during the whole session of experiments. Before starting each experimental session, rats were accustomed to various handling procedures in order to avoid any stress effect and to nullify the psychological affliction of the environment. All the experiments were performed ethically in accordance with the guidelines of the Local Animal Care Ethical Committee.

### 2.2. Experimental Protocol

Rats were divided into two groups: control and test. Control rats received water while to the test group olive oil was given at the dose of 0.25 mL/kg body weight daily for 27 days. On the 28th day, behavioral analysis was performed. Behavioral tests include plus maze activity and forced swim test. Rats were then decapitated, and their brain was removed within 30 seconds from skull. The membrane covering the brain was removed with the help of fine forceps. The brain then taken out using spatula was dipped in ice-cold saline. All samples were stored at −70°C until neurochemical analysis was done. Frozen brain samples were homogenized in extraction medium using an electrical homogenizer, and neurochemical analysis was performed to estimate concentrations of 5-HT (5-hydroxytryptamine) and its metabolite 5-HIAA (5-hydroxyindoleacetic acid) and DA (dopamine) and its metabolite HVA (homovalinic acid) in the whole brain of rats by HPLC-EC method as reported by Haider et al. 2004 [22]. A 5 *μ* Shim-pack ODS separation column of 4.0 mm internal diameter and 150 mm length was used as the stationary phase. Separation was achieved by a mobile phase containing methanol (14%), octyl sodium sulfate (0.023%), and EDTA (0.0035%) in 0.1 M phosphate buffer at pH 2.9 at an operating pressure of 2000–3000 psi on Schimadzu LEC 6A detector at an operating potential of 0.8 volts for biogenic amines.

### 2.3. Behavioral Tests

#### 2.3.1. Elevated Plus Maze Test (EPM)

Elevated plus maze (EPM) is an apparatus used to evaluate the rodent's natural behavior which is fear of novel and open areas. The anxiolytic activity of drug in elevated plus maze model was measured according to method as previously reported [23]. Plus maze apparatus consists of four equal size arms. The two opposite arms are open while two were closed. The length of each arm was 50 cm and width is 10 cm. Arms were joined by the central area of 5 cm^2^. The length of the wall of the closed arm was 40 cm. The maze was elevated from ground at a height of 60 cm. To determine activity, a rat was placed in the center of the plus maze and the time spent in the open arm was monitored for 5 minutes. 

#### 2.3.2. Forced Swimming Test

Assessment of depressive symptoms was monitored by forced swim test (FST) following 4 weeks of oral administration of drug. FST was performed as described by Detke et al. [24]. To monitor the antidepressant activity, rats are placed individually in a tank (53, 19, 28 cm). The water is filled up to 18 cm. The height of the water is such that animal is supposed to swim. The animal is subjected in the container for 5 minutes, and behavioral scoring was performed by noting immobility time. After each test, rats were dried with towel and placed in home cage. 

### 2.4. Statistical Analysis

The data was analyzed by Student's *t*-test. Values are represented as mean ± SD, and values of *P* < 0.05 are considered as significant.

## 3. Results

**Figure 1:**
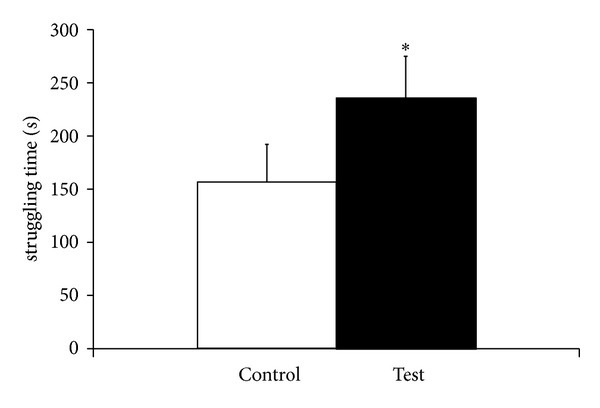
Effect of repeated administration of olive oil on struggling time in force swim test. Values are mean ± SD (*n* = 5) significance difference by Student's *t*-test *(*P* < 0.05) saline treated controls.

Figure 1 shows the effect of repeated administration of olive oil on depression like symptoms in animals. Student's *t*-test analysis showed that there is a significant (*P* < 0.05) increase in struggling time following repeated administration of extravirgin olive oil indicating that olive oil has significant antidepressant effects.

**Figure 2:**
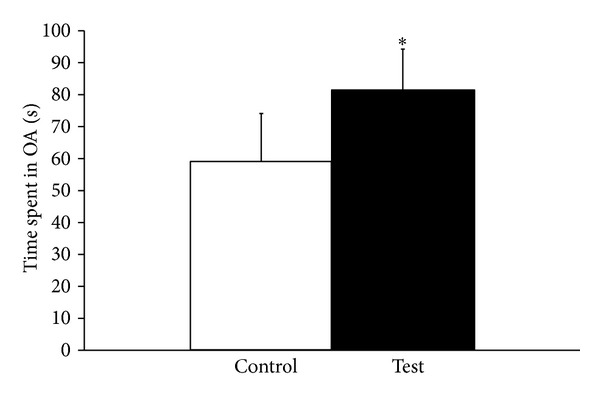
Effect of repeated administration of olive oil on time spent in open arm in elevated plus maze model. Values are mean ± SD (*n* = 5) significance difference by Student's *t*-test *(*P* < 0.05) saline treated controls.

Figure 2 shows the effect of olive on anxiety in animals. Analysis by Student's *t*-test revealed that there was a significant (*P* < 0.05) increase in time spent in open arm following repeated administration of extra-virgin olive oil. This indicates that administration of olive oil produces anxiolytic effects in animals. 

Table 1 shows the effect of olive oil on levels of brain 5-HT and its metabolite 5-HIAA. Student's *t*-test analysis showed a significant (*P* < 0.05) decrease in brain 5-HT and 5-HIAA levels following repeated administration of extra-virgin olive oil which indicates a decrease in 5-HT metabolism and synthesis. 

Table 2 shows the effect of repeated administration of olive oil on levels of brain DA and its metabolite HVA. Analysis by Student's *t*-test revealed that there was a significant (*P* < 0.05) decrease in brain DA levels while the HVA levels were significantly (*P* < 0.01) increased following repeated administration of extra-virgin olive oil which indicates that olive oil administration led to an enhancement in HVA levels and a decrease in brain DA by increasing DA metabolism.

## 4. Discussion

Olive oil is used throughout the world mostly in the mediterranean because of its beneficial health effects. In the last few years, the number of reports describing the beneficial properties of olive has been increasing. Recent data has suggested that component of olive oil may have more health benefits than previously thought. The present study reveals that repeated administration of extra-virgin olive oil produces anxiolytic and antidepressant effects via neurochemical alterations in brain 5-HT, DA, and their metabolites. 

Role of omega-3 fatty acid in adaptation to stress has been documented previously [13]. Reports have shown that low levels of omega-3 and omega-6 fatty acids are involved in producing depression as omega-3 fatty acids are important component of nerve cell membrane and help nerve cells to communicate with each other, which is an essential step in maintaining good mental health. Omega-3 fatty acid of DHA group has been shown to decrease or prevent aggression during examination [25]. High levels of omega-3 fatty acid in serum have been shown to lower the levels of pro-inflammatory cytokines thus protecting from stress [26]. In the present study, it is has been observed that repeated administration of extra-virgin olive oil produce significant antidepressant effects as the struggling time of rats taking olive oil was significantly increased as compared to control rats. This indicates that olive oil has great potential to reduce depression which may be due to presence of linoleic acid; a polyunsaturated omega-3 fatty acid that makes up 1.5% of extra-virgin olive oil [27].

Studies on animal model showed an association of depressive behavior with altered DA function [28]. Evidence from animal and human studies showed that DA and NA play important role in pathophysiology as well as in therapeutic effect of antidepressants [29]. Evidence from clinical studies supports the finding that depressed patients have reduced CSF (cerebrospinal fluid) levels of HVA [30]. Decreased DA and increased levels of HVA are an indication of increased release of DA and increased turnover which may play important role in antidepressant effect of DA [31]. Decreased DA and increased HVA levels together with the increased struggling time following repeated administration of extra-virgin olive oil observed in the present study support the findings that olive oil produces antidepressive effect by increasing the release and turnover of DA.

The present findings also revealed that the anxiolytic property of extra-virgin olive oil as time spent in open arms in EPM was increased significantly following repeated administration of extra-virgin olive oil. The anxiolytic effect of extra-virgin olive oil could be due to presence of phenolic antioxidants, olieic acid, tocopherols, and oleuropin in olive oil. It has been reported that phenolic antioxidants produce anxiolytic effect [18]. Anxiolytic effects of olive oil observed in the present study may be due to alterations in brain 5-HT metabolism. Role of 5-HT in anxiety is well documented. Increased 5-HT produces anxiogenic effect while decreased level of 5-HT produces anxiolytic effect [19]. In the present study, it has been found that extra-virgin olive oil decreases brain 5-HT as well as 5-HIAA levels in rat brain. Administration of extra-virgin olive oils ignificantly decreases 5-HT synthesis and metabolism that may play important role in reducing anxiety in animals. Decreased 5-HT and 5-HIAA levels together with the increased time spent in open arm in EPM observed in present study indicate that extra-virgin olive oil produces anxiolytic effect by decreasing 5-HT metabolism.

## 5. Conclusion

Olive oil is an essential ingredient of Mediterranean food. Olive oil has important effect on body and has protective effect against several pathological conditions. Results of present study show that olive oil produces anxiolytic effect via decreasing brain 5-HT synthesis and metabolism. However, antidepressive effect of olive oil may attribute to the increased DA release and its metabolism. Results of the present study supported the use of olive oil as food supplementation for mood elevation.

## Figures and Tables

**Table 1:** Effect of repeated administration of olive oil on brain 5-HT levels and its metabolite 5-HIAA levels.

Parameter	Control (water)	Test (olive oil)	Significance
5-HT	52.6 ± 13.5	31 ± 4.6	**P < 0.01
5-HIAA	97.2 ± 6.8	68.2 ± 7.9	**P < 0.01

Values are mean ± SD (*n* = 5) and significant differences by Student's *t*-test are represented as **(*P* < 0.01) from water treated control.

**Table 2:** Effect of repeated administration of olive oil on brain DA levels and its metabolite HVA levels.

Parameter	Control (water)	Test (olive oil)	Significance
DA	309.6 ± 17.3	273.4 ± 68.3	*P < 0.05
HVA	50.8 ± 7.8	139.6 ± 14.9	**P < 0.01

Values are mean ± SD (*n* = 5) and significant differences by Student's *t*-test are represented as **(*P* < 0.01) and *(*P* < 0.05) from water treated control.
